# Post-traumatic stress disorder symptom burden and gender each affect generalization in a reward- and punishment-learning task

**DOI:** 10.1371/journal.pone.0172144

**Published:** 2017-02-14

**Authors:** Milen L. Radell, Kevin D. Beck, Mark W. Gilbertson, Catherine E. Myers

**Affiliations:** 1 Department of Psychology, Niagara University, Lewiston, NY, United States of America; 2 Department of Veterans Affairs, New Jersey Health Care System, East Orange, NJ, United States of America; 3 Department of Pharmacology, Physiology & Neuroscience, New Jersey Medical School, Rutgers University, Newark, NJ, United States of America; 4 Department of Veterans Affairs, Manchester, NH, United States of America; Yale University, UNITED STATES

## Abstract

Post-traumatic stress disorder (PTSD) can develop following exposure to a traumatic event. Re-experiencing, which includes intrusive memories or flashbacks of the trauma, is a core symptom cluster of PTSD. From an associative learning perspective, this cluster may be attributed to cues associated with the trauma, which have come to elicit symptoms in a variety of situations encountered in daily life due to a tendency to overgeneralize. Consistent with this, prior studies have indicated that both individuals with clinically diagnosed with PTSD, and those with self-reported symptoms who may not meet full diagnostic criteria, show changes in generalization. Building on prior research, the current study examined whether PTSD symptom burden, but also gender, veteran status, and combat experience–all associated with PTSD vulnerability–modulate learning and generalization in a computer-based task. Participants were presented with stimulus compounds consisting of a foreground and background that could be predictive of reward, punishment or no outcome. Learning was followed by a generalization test where these components were recombined to form novel configurations. An interaction between PTSD symptom burden and gender was found where females with more severe PTSD symptoms showed no evidence of sensitivity to the background. This result is consistent with increased generalization, and may indicate a decrease in the ability to process cue configurations leading to re-experiencing in a variety of situations. Further work is indicated to help elucidate the cognitive processes driving gender differences that may confer vulnerability to PTSD.

## Introduction

Post-traumatic stress disorder (PTSD) is a syndrome that can develop in the wake of exposure to a traumatic event. Symptoms include re-experiencing (intrusive recollection, nightmares, and flashbacks), physiological arousal, emotional numbing, and cognitive and behavioral avoidance. Although most individuals experiencing a traumatic event will not develop PTSD, prevalence is quite high in some populations. For example, lifetime prevalence of PTSD among adult Americans has been estimated to be about 6.8%, but prevalence may be more than twice as high among women than men [1]. Another population at high risk is veterans. While military personnel may have the same vulnerability to PTSD as the general population, the extreme stressors of deployment, war, and wartime service place veterans at greater risk for developing PTSD. One study estimated that about 15–20% of military personnel returning from combat duty in Afghanistan or Iraq met PTSD criteria 3–4 months later [2], while a recent re-examination of data on Vietnam-era veterans with theater service found a lifetime PTSD prevalence of about 20% [3].

The impact of PTSD is significant, and includes health and personal costs for the individual, his or her family, and society. These costs can include difficulty maintaining employment, family disruption, and co-morbid alcohol and drug abuse. Further, in addition to clinically-diagnosed PTSD, some individuals exposed to a traumatic event develop subclinical PTSD, presenting with at least one symptom but falling short of full diagnostic criteria. Prevalence of subclinical PTSD among those exposed to traumatic events may be as high as 70–80% [4], and subclinical PTSD confers heightened risk for subsequent conversion to full-blown PTSD [5], as well as increased risk for depression, alcohol abuse, poor health, and disability following physical injury [6–8]. Fewer studies, however, have included subclinical cases despite the cost to affected individuals.

A defining feature of PTSD is re-experiencing symptoms, which include intrusive memories or flashbacks of the trauma [9]. Re-experiencing can be explained from an associative learning perspective, where a variety of neutral cues present at the time of trauma become associated with this event, and come to elicit fear. An increased tendency to generalize this learning to other stimuli may lead to re-experiencing even in situations that pose no actual danger [9, 10].Changes in generalization have also been used to explain re-experiencing in computational accounts of PTSD [11]. Interestingly, these changes may not be limited to trauma-related stimuli. This is supported by prior studies, most of which have documented increased generalization in individuals with more severe PTSD symptoms, even using neutral stimuli in computer-based associative learning tasks [12–14]. A notable exception is a study that instead reported reduced generalization in patients with PTSD compared to non-PTSD controls [15].

A tendency to overgeneralize may reflect loss of contextual information or changes in how compound stimuli are processed, allowing for previously-neutral cues associated with the experience of a traumatic event to elicit re-experiencing, even in novel situations encountered over the course of daily life, where these cues may be ubiquitous. In a particularly relevant study for this issue by Levy-Gigi et al. [14], participants with and without PTSD were trained on an associative learning task where they could choose to “open” or “skip” (leave closed) stimuli in the form of boxes with a foreground object presented against a colored background (or “context”). Two stimuli were positively-valenced (e.g. “open” responses triggered point gain) and two stimuli were negatively-valenced (i.e. “open” responses triggered point loss), while “skip” responses triggered no points gained or lost. Training was followed by a transfer phase that included “retention” trials with trained foreground-background configurations, “foreground reversal” trials where a familiar foreground was presented against a novel background and given opposite valence from the training phase, and “background reversal” trials where a familiar background was paired with a novel foreground and given opposite valence from the training phase. No group effects on training, retention, or foreground reversal trials were found. However, the PTSD group was selectively impaired at background reversal involving a negative background [14]. This prior study, like many others, was underpowered to examine gender effects, despite the fact that women appear to be at higher risk for PTSD than men [16] and the course and expression of PTSD may be different in females than in males [17]. In addition, the use of explicit reversal training, with results reported as an average across the entire transfer phase, means that the prior results were unable to decouple generalization biases from learning speed.

The purpose of the current study was to examine reward- and punishment-based associative learning and generalization in a large group of participants, including males, females, veterans and civilians, in a similar task but without reversal training. In contrast to the previous study by Levy-Gigi et al. [14], individuals with subclinical symptoms who may not meet criteria for a formal PTSD diagnosis, were also included in the current sample. Participants were first trained to “pick” two positively-valenced foreground-background combinations and to “skip” two negatively-valenced combinations. Subsequently, participants were tested, without feedback, to assess generalization on all possible combinations of positive, negative, and novel foregrounds paired with positive, negative, and novel backgrounds. Generalization was assessed both behaviorally (via “pick” vs. “skip” responses) as well as by asking participants to quantify their confidence in these responses. We examined, first, whether PTSD symptom burden differentially affects reward- and punishment-based learning; second, whether there are effects of gender and military service; and, third, whether any of these between-subject variables affect generalization. Consistent with most prior studies, we predicted that individuals with severe PTSD symptoms will show increased generalization, and that changes in generalization should be particularly evident in women, regardless of military service.

## Methods

### Participants

A total of 141 participants, including 90 veterans and 51 civilians (never served in the military), were recruited from the East Orange VA Medical Center and surrounding community by flyer and word-of-mouth referral. An additional 22 participants were recruited and used as a pilot to estimate effect size and permit *a priori* power analysis to determine required sample size (see Data Analysis below). To avoid circularity in the power analysis (i.e. avoid double-dipping), data from the 22 pilot participants were not included in the final sample or dataset. All subjects were paid $20/hour for their participation in a single 2-hour session and signed statements of informed consent before the start of any testing. Study procedures were approved by the Institutional Review Board at the VA New Jersey Health Care System and conformed to guidelines established by the Declaration of Helsinki and the United States Federal Government for the protection of human subjects.

Because males were overrepresented in the veteran sample (about 85% male), females were intentionally oversampled in the civilian sample to adequately power the study to examine gender as well as PTSS and military service (see Data Analysis below). Additionally, as described below, the veteran group was subdivided post-hoc on the basis of history of exposure to combat, creating three participant groups (civilian, non-combat veteran, and combat veteran).

One veteran participant withdrew from the study prior to completing the tasks–this individual’s data were excluded from subsequent analysis. Three additional participants (two veterans, one civilian) were excluded post-hoc due to possible partial colorblindness, assessed via the Ishihara colorblindness test [18], since abnormal color vision might affect both the ability to perceive colors on the computer screen, and the degree to which a participant would use colored backgrounds to modulate responding to foreground objects.

The resulting set of 137 participants (87 veterans, 50 civilians) included 86 males and 51 females, with a mean age and education of 52.1 years (SD 12.4) and 15.0 years (SD 2.7), respectively. One civilian subject declined to specify years of education. Asked to self-report race and ethnicity, 91 identified as Black, African, or African-American, 23 as White or Caucasian, 1 as Native American, 11 as Other, and 10 as mixed race. One individual declined to specify race. Finally, when asked to self-identify ethnicity as Hispanic or non-Hispanic, six participants identified as Hispanic and two as “unsure.”

Veteran participants were also asked about specific conflicts in which they had served; 30 specified having served in Vietnam, 14 in Gulf War/Operation Desert Storm, 13 in Operation Enduring Freedom/Operation Iraqi Freedom, 9 in other conflicts (e.g. Korea, Bosnia, Somalia, Beirut/Lebanon), and 29 reported no specific conflict or peacetime service. Numbers sum to greater than 85 due to some veterans whose service spanned multiple conflicts.

### Procedure

Testing took place in a quiet room with the participant seated at a comfortable viewing distance from the computer for the contextual generalization task, or at a comfortable writing position to complete the questionnaires. The experimenter remained present in the room throughout the testing session but, after delivering instructions for each task, withdrew to a separate table in the room.

All participants received a short demographic questionnaire that included questions about age, gender, education, and military service. Participants also received the Posttraumatic Stress Checklist (PCL-C), a 17-item self-report questionnaire that asks about presence and frequency of PTSD symptoms not necessarily military in nature [19]. PCL questions correspond to specific PTSD symptom clusters as defined by the revised 4^th^ edition of the Diagnostic and Statistical Manual of Mental Disorders (DSM-IV) [20], including re-experiencing, avoidance/numbing, and increased arousal. PCL scores of 50+ have been shown to predict PTSD in military samples [19, 21]. Thus, we classified participants according to the presence or absence of current, self-reported, severe PTSD symptoms (PTSS) based on this cutoff.

Veteran participants also received the Combat Exposure Scale (CES), which assesses exposure to stressful military events [22]. Following prior studies [23, 24], veterans scoring 8 or higher on the CES were classified as having a history of exposure to combat, and those scoring below 8 were classified as non-combat-exposed.

Participants then completed the generalization task described below. Finally, participants were administered a colorblindness test after the generalization test to avoid biasing their responses to colored stimuli. As permitted within the constraints of the 2-hour testing session, some participants also completed additional tasks (e.g. piloting of new behavioral tasks or questionnaires)–these data are not reported here.

#### Generalization task

The generalization task, which took about 10 minutes to complete, was presented on a Macintosh computer programmed in the SuperCard language (Solutions Etcetera, Pollock Pines, CA). In an initial training phase, subjects learn through trial and error to “pick” or “skip” each of four compound stimuli. Each stimulus is composed of a white geometric shape, serving as the foreground, presented against a colored background. The four foregrounds in the training phase were randomly selected for each participant from a set of seven shapes (triangle, heart, star, parallelogram, circle, diamond, square), and the four backgrounds were randomly selected from a set of seven distinct colors (green, purple, yellow, pink, brown, blue, red). Two stimuli were positively-valenced, meaning that a “pick” response resulted in point gain, and two stimuli were negatively-valenced, meaning that a “pick” response resulted in point loss. “Skip” responses to either type of stimulus triggered an acknowledgment of the response, but no point gain or loss. The training phase was followed by a generalization test, in which subjects were presented with new stimuli consisting of positive, negative, or new foregrounds presented against positive, negative, or new backgrounds.

**Fig 1:**
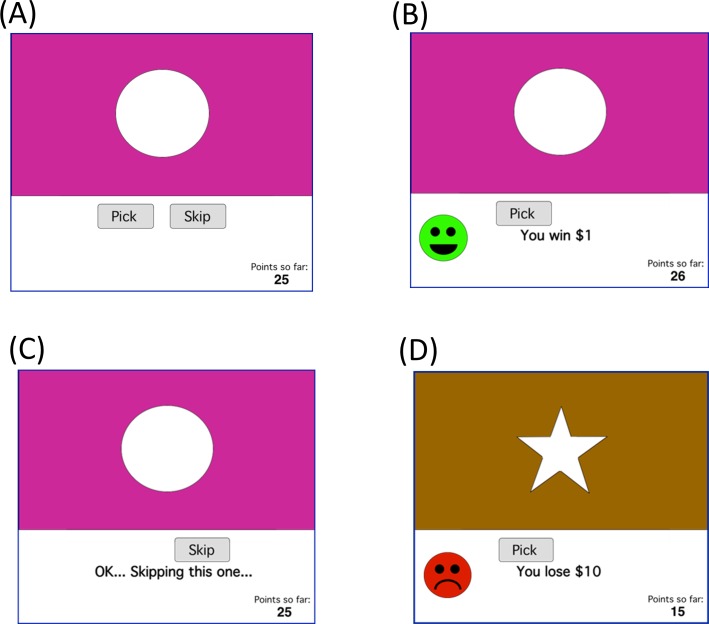
Example screen events during the training phase. (A) At the start of a trial, the screen shows one of four stimuli, each composed of a white geometric shape presented against a colored background. The subject was prompted to “pick” or “skip” this stimulus by using the mouse to click on the on-screen buttons. (B) On reward-based trials, a positively-valenced stimulus was presented, and a “pick” response resulted in positive feedback and point gain; (C) a “skip” response resulted in no point gain (or loss). (D) On punishment-based trials, a negatively-valenced stimulus was presented, and a “pick” response resulted in negative feedback and point loss, while a “skip” response resulted in no point change.

At the start of the experiment, participants saw an instruction screen that read: *“On each trial in this experiment*, *you will be given a choice whether you want to “pick” or “skip*.*” If you “pick*,*” you might win some money–or you might lose some money*. *If you “skip*,*” you won’t win or lose any money*. *Your job is to collect as much money as you can*. *We’ll start you off with a little money to begin with*.*”* Backgrounds appeared about 4” high and 8” wide on the screen, and foregrounds appeared about 2” high, centered within the background. Fig 1A shows an example screenshot from one training trial. Two onscreen buttons, labeled “pick” and “skip,” appear below the stimulus, and the participant can use the mouse to click on one of these buttons to register a response. The point tally was initialized to 25 at the start of the experiment and remained visible onscreen throughout training.

On reward-based trials, a positively-valenced stimulus appeared. If the participant responded “pick,” a green smiley face appeared with the legend “*You win $1*!” and the point tally increased by 1 (Fig 1B). On punishment-based trials, a negatively-valenced stimulus appeared–a “pick” response was followed by the appearance of a red frowning face with the legend “*You lose $10*” and the point tally decreased by 10 (Fig 1D). The magnitude of punishment was greater than that of reward to promote making careful choices in the task, and discourage making random guesses when skipping the trial would be more appropriate. On either type of trial, “skip” responses triggered an acknowledgment, “*OK*, *skipping this one*…” but did not result in point gain or loss. Feedback remained visible for 2 seconds. The training phase continued for 64 trials, including 4 blocks of 16 trials, with each block containing 4 presentations of each of the 4 stimuli, in random order.

**Fig 2:**
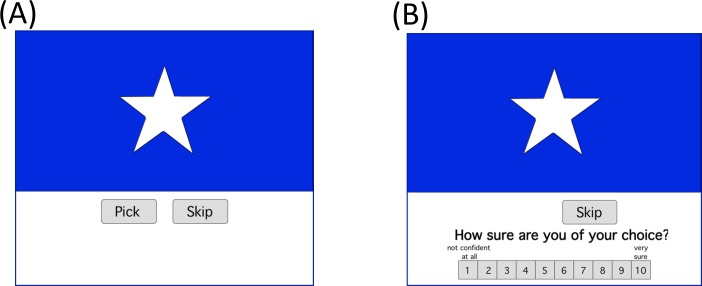
Sample screen events during a test trial. (A) Here, a negative object (star) is presented against a new context (blue), and the participant is asked to “pick” or “skip”. (B) After each response, the participant is asked to rate confidence in this choice on a Likert scale from 1 =“not confident at all” to 10 =“very sure.” No feedback is provided during the testing phase.

Following the training phase, a test phase began, with new instructions on the screen: “*So far so good*. *Now*, *you should continue to pick and skip*, *but you won’t be shown whether you were correct or not*. *At the very end we’ll tell you how much total money you won*.” Trials in the testing phase were similar to those in the training phase, except no feedback was provided (and no points were won or lost), and following each “pick” or “skip” response, subjects were asked to rate their confidence in their response by clicking onscreen buttons representing a Likert scale that ranged from 1 =“not confident at all” to 10 =“very sure” (Fig 2). Trials in the testing phase involved nine new stimuli. Each consisted of a positive, negative, or new foreground presented against a positive, negative, or new background. No foreground-background configurations that had appeared during training were presented during testing. The test phase included 3 blocks of trials, each with one presentation of the 9 test stimuli in random order.

#### Data analysis

*A priori* power analysis was conducted after a pilot phase, which allowed computation of estimated effect sizes. The power analysis (using G*Power v. 3.0.10) indicated that a sample consisting of approximately 50 civilians, 50 non-combat veterans, and 30 combat veterans, and composed of approximately 80 males and 50 females, with about 50% PTSS cases, should be adequately powered to detect significant effects, if they exist (Group: estimated effect size f = .24, power>80% with alpha = .05, two-tailed; Gender and PTSS: both estimated effect size f>.42; both power>80% with alpha = .05, two tailed). Data collection was halted when these recruitment targets were met (see also Table 1 below).

The primary analyses for the computer-based task were mixed ANOVA on the dependent measures, with between-subjects factors of group (civilian, non-combat veteran, combat veteran), gender (male, female), and PTSD symptom severity (noPTSS, PTSS). For the training phase, dependent measures were percent correct, using within-subjects factors of trial type (reward- vs. punishment-based) and training block (4 blocks). A similar analysis was conducted on percent “pick” responses to examine possible response bias. For the testing phase, dependent measures were percent “pick” responses, using within-subjects factors of foreground valence (positive, negative, new) and background valence (positive, negative, new). A similar analysis was also conducted on confidence ratings.

Threshold for significance was set at alpha = .05. Where omnibus tests were significant and multiple post-hoc tests were conducted to explore significant results, Bonferroni correction was used to adjust significance thresholds to protect against Type I error. Statistics for tests that fell short of corrected significance, but would be significant at the uncorrected alpha = .05 are still reported (although not considered significant). Where the data failed statistical assumptions for parametric tests (e.g. assumptions of sphericity and equal variance) appropriate corrections were used to adjust degrees of freedom (e.g. Greenhouse-Geisser for ANOVA, Welch’s *t* for *t*-test). In all figures showing bar or line graphs, error bars represent standard error of the mean (SEM).

## Results

### Questionnaire results

Based on CES scores, 33 of the 87 veteran participants were classified as combat-exposed and the remaining 54 as non-combat-exposed. This resulted in three participant groups, as summarized in Table 1. The three groups did not differ in age (*F*(2,134) = 2.86, *p* = .061) or education (*F*(2,134) = 2.27, *p* = .107). The difference in gender distribution was significant (χ^2^(2) = 38.16, *p* < .001), with only 3 females in the combat-exposed group. In contrast, over half of the civilian group was female.

**Table 1:** Demographic information and PCL scores for the veteran and civilian groups.

	Civilian	Non-combat veteran	Combat veteran
N	50	54	33
Gender (% female)	35 female (70.0%)	13 female (24.1%)	3 female (9.1%)
Age in years	50.5 (SD 13.4)	55.2 (SD 9.9)	49.6 (SD 13.8)
Education in years	15.2 (SD 3.0)	14.5 (2.3)	15.6 (SD 2.7)
PCL	37.8 (SD 13.7)	52.9 (SD 16.0)	57.1 (SD 18.4)
PTSS cases	11 PTSS (22.0%)	32 PTSS (59.3%)	25 PTSS (75.8%)

Table 1 also shows mean PCL scores for each group. ANOVA confirmed a significant effect of group (*F*(2,131) = 12.54, *p* < .001) with no main effect of gender (*F*(1,131) = 0.01, *p* = .925) but a significant gender-group interaction (*F*(2,131) = 5.60, *p* = .005). Tukey’s HSD confirmed significantly lower PCL scores in the civilian group than in either of the veteran groups (both *p* < .001) but the combat and non-combat veteran groups did not differ (*p* = .430).

Pairwise independent *t*-tests were also conducted to compare PCL scores in males vs. females across the three groups (alpha adjusted to .05/3 = .0167). In the civilian group, males had higher PCL scores than females (males 46.7, SD 13.7, females 33.7, SD 11.8, *t*(48) = 3.39, *p* = .001). In non-combat veterans, a trend for higher scores in females fell just short of corrected significance (males 50.6, SD 17.0; females 59.9, SD 9.4; Welch’s *t*(37.8) = 2.51, *p* = .017). In combat veterans, there was no gender difference (males 56.6, SD 19.0, females 61.3, SD 12.5, *t*(31) = 0.42, *p* = .680), although the lack of significance may simply reflect the low number of females in this group.

Based on PCL scores, 68 participants (49.6%) met criteria for PTSS. The distribution of PTSS cases differed significantly across groups (χ^2^(2) = 26.28, *p* < .001), with fewer cases among civilians than either veteran group (both Yates-corrected χ^2^(1)>14.00, both *p* < .001); however, rates did not differ between the two veteran groups (Yates-corrected χ^2^(1) = 2.47, *p* = .116).

### Generalization task: Training phase

Data from two participants were dropped based on apparent noncompliance during the training phase, including one participant who simply responded “pick” on all 64 trials, and one who responded “skip” on all of the last 48 trials. Data from an additional seven participants were dropped based on test phase data, including five participants who responded “pick” to 100% of trials during the test, and two who responded “skip” to 100% of trials during the test. These nine apparently non-compliant participants included 4 combat veterans, 2 non-combat veterans, and 3 civilians. The remaining sample of *n* = 128 participants was used for all subsequent analyses. The values of the means and standard errors used to construct all data figures are included in the S1 File.

**Fig 3:**
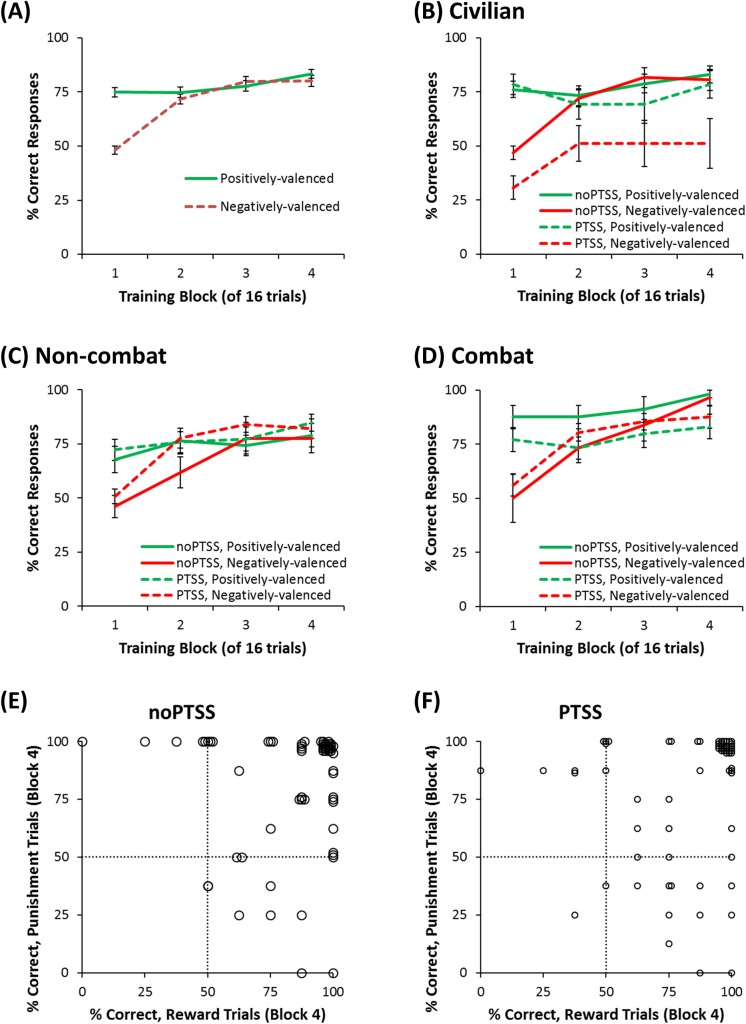
Training phase data from the generalization task. (A) Across blocks, participants increased performance, measured as percent correct responding on reward-based and punishment-based trials. (B-D) Within the three groups, only in the civilian group did the difference between PTSS and noPTSS participants approach corrected significance. (E, F) By training block 4, all but one noPTSS participant and two PTSS participants were responding above-chance (50%) on reward-based or punishment-based trials, although not all participants were equally accurate on both. Note some data points are jittered slightly in (E) and (F) for clarity.

Fig 3A shows that percent correct increased across the four training blocks (*F*(2.62, 304.43) = 42.11, *p* < .001). There was also an effect of trial type (*F*(1,116) = 8.23, *p* = .005) and a block-type interaction (*F*(2.54,294.66) = 17.42, *p* < .001). Specifically, participants learned reward-based associations faster than punishment-based associations. There was no main effect of gender (*F*(1,116)<0.01, *p* = .947) nor any interactions involving gender (all *p*>.100); however, there was a significant group-PTSS interaction (Fig 3B–3D; F(2,116) = 3.47, p = .034). To investigate this interaction, independent-samples *t*-tests were conducted to compare total percent correct responding among those with vs. without PTSS in each of the three groups. In civilians, the difference between PTSS and noPTSS approached, but fell short of, corrected significance (*F*(1,45) = 6.19, *p* = .017). There was no difference between PTSS and noPTSS participants among the two veteran groups (all *F*<1.5, all *p*>.200). By block 4, all participants except one in the noPTSS group, and two in the PTSS group, were performing above chance (50%) on reward-based trials, punishment-based trials or both (Fig 3E and 3F).

**Fig 4:**
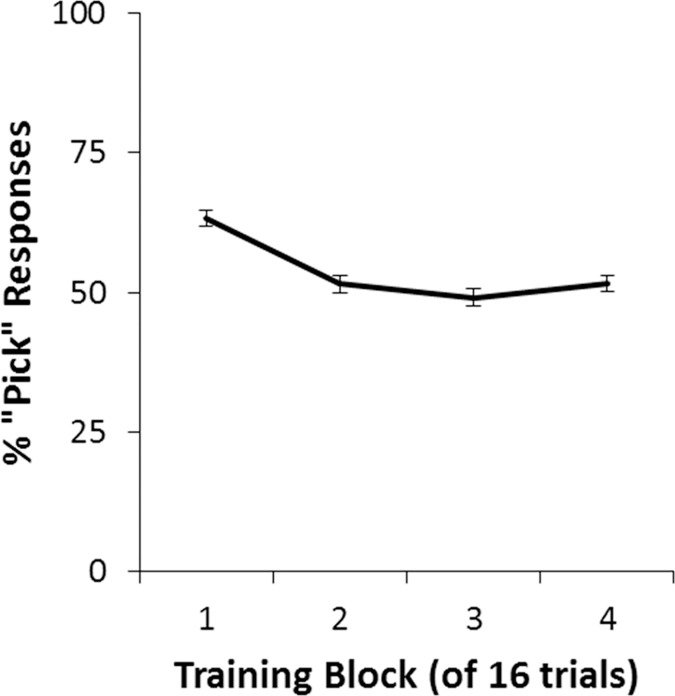
Percent “pick” responses per block in the training phase. Although participants tended to make “pick” responses on about two-thirds of trials in the first block of training, the response rate approached 50% in later blocks (dashed line), indicating a balance between “pick” and “skip” responses.

Percent “pick” responses also changed across the four blocks (Fig 4; *F*(2.54, 294.66) = 17.42, *p* < .001), with no between-subjects effects or interactions (all *p*>.100). Specifically, although participants initially tended to make “pick” responses on about two-thirds of trials in the first block, the response rate approached 50% in later blocks, which would be the theoretical optimum if participants were responding correctly to all trials.

### Generalization task: Testing phase

**Fig 5:**
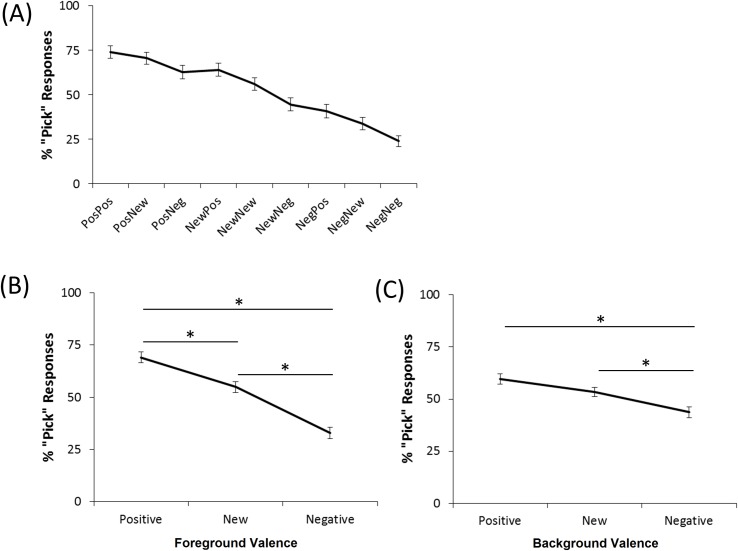
Results from the testing phase of the generalization task. (A) Participant responding, indexed as percent “pick” responses over the three trials with each test stimulus, was modulated both by foreground and background valence. Labels along the x-axes are of the form “foreground-background” indicating whether the foreground or background was positive (“Pos”), previously negative (“Neg”) or New. For example, “PosPos” indicates a positive foreground paired with a positive background, while “PosNew” indicates a positive foreground paired with a new background that was not experienced during the training phase. (B) Responding was modulated by foreground valence, with more “pick” responses to stimuli with positive than new foregrounds, regardless of background. Both positive and new foregrounds were picked more often than negative foregrounds. (C) Similarly, background valence affected responding with more “pick” responses to stimuli with either positive or new backgrounds compared to those with negative backgrounds. Asterisks indicate significant within-subject differences.

Fig 5 shows participants tended to make more “pick” responses to stimuli composed of positive, compared to negative, foregrounds and backgrounds. Mixed ANOVA confirmed these impressions, with significant main effects of foreground (*F*(2,232) = 21.52, *p* < .001) and background (*F*(1.76, 204.57) = 17.17, *p* < .001), but no foreground-background interaction (*F*(4, 464) = 0.50, *p* = .734). Post-hoc paired-samples *t*-tests (with alpha adjusted to .05/6 = .0083 to protect significance) to examine the main effect of foreground confirmed that participants tended to “pick” stimuli with a positive foreground more often than those with a new foreground (paired-samples *t*-test, *t*(127) = 4.28, *p* < .001), and to pick both of these more often than stimuli with a negative foreground (both *t*>6, both *p* < .001). Post-hoc tests to examine the main effect of background confirmed that subjects were significantly less likely to “pick” stimuli with a negative background than either a positive or novel background (both *t*>3.5, both *p* < .001). There was also a trend for subjects to “pick” stimuli with a foreground presented against a positive background more often than those presented against a new background, but this did not reach corrected significance (*t*(127) = 2.43, *p* = .017). Thus, the task successfully elicited both foreground- and background-modulated responding.

**Fig 6:**
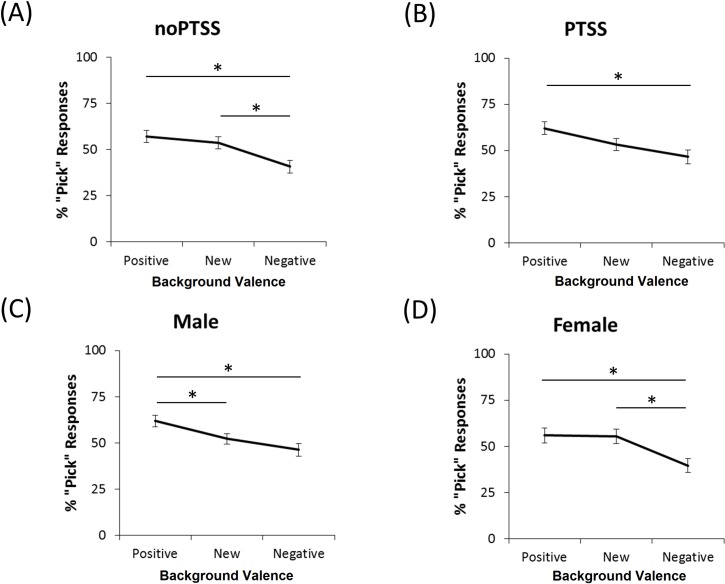
Percent “pick” responses as a function of PTSS and gender. (A) Generally, “pick” responses were made on about 50% of trials with a new background. However, participants in the noPTSS group tended to treat positive and new backgrounds as similar, and make fewer “pick” responses on trials with a negative background. (B) For participants in the PTSS group, only the difference between positive and negative backgrounds reached significance. (C) While males tended to make more “pick” responses for positive backgrounds, compared to either new or negative backgrounds, (D) females did not distinguish positive from new backgrounds. Instead, they made more “pick” responses to new compared to negative backgrounds. Similar to males, they also made more “pick” responses for positive than negative backgrounds. Asterisks indicate significant within-subject differences.

The omnibus ANOVA also revealed several significant between-subjects effects. First, there was a two-way interaction between background valence and PTSS (*F*(1.76, 204.57) = 5.28, *p* = .008). Post-hoc paired-samples *t*-tests were run separately for PTSS and noPTSS groups (Fig 6A and 6B). The noPTSS group made fewer “pick” responses in a negative, compared to a positive or new, background (all *t*>3.4, all *p* = .001) but rates of “pick” responding in positive and new backgrounds did not differ (*t*(61) = 1.00, *p* = .323). Similarly, the PTSS group responded “pick” more often in a positive than negative background (*t*(65) = 3.37, *p* = .001) but did not distinguish new from either positive or negative backgrounds (all 2<*t*<2.5, all .0167<*p* < .050). Direct comparisons via Bonferroni-corrected independent-samples *t*-tests between the noPTSS and PTSS groups at each background valence were also run (Bonferroni-corrected alpha = .05/3 = .0167). No contrasts were significant, including those for the positive, *t*(126) = 0.98, *p* = .330, new, *t*(126) = 0.05, *p* = .962, and negative, *t*(126) = 1.17, *p* = .246, background.

A specific prediction from the prior Levy-Gigi et al. [14] study was that the PTSS group should show fewer “pick” responses than the noPTSS group when a new foreground was presented against a negative background, but not when a new foreground was presented against a positive background. However, neither comparison was significant in the present data set (independent-samples *t*-tests, all *t*<2, all *p*>.080).

The omnibus ANOVA also revealed a two-way interaction between background valence and gender (*F*(1.76, 204.57) = 5.70, *p* = .006). Post-hoc paired-samples *t*-tests were run separately for males and females (Fig 6C and 6D). Males made significantly more “pick” responses to foregrounds in positive backgrounds than in new or negative backgrounds (all *t*>2.8, all *p*< = .005). However, there was no difference between new and negative backgrounds (*t*(78) = 1.88), *p* = .064). Females made significantly less “pick” responses to foregrounds in a negative than in either a new or positive background (all *t*>3.2, all *p*< = .002). In contrast to males, they did not distinguish positive from new backgrounds (*t*(48) = 0.22, *p* = .825). In short, while both genders picked objects in new backgrounds at a rate of about 50%, males were more likely to pick objects in a positive background, and females were less likely to pick objects in a negative background. Bonferroni-corrected independent-samples *t*-tests comparing males and females at each background valence were also run (Bonferroni-corrected alpha = .05/3 = .0167). There were no significant differences for either the positive, *t*(126) = 1.08, *p* = .281, new, *t*(126) = 0.72, *p* = .474, or negative, *t*(126) = 1.26, *p* = .212, background.

**Fig 7:**
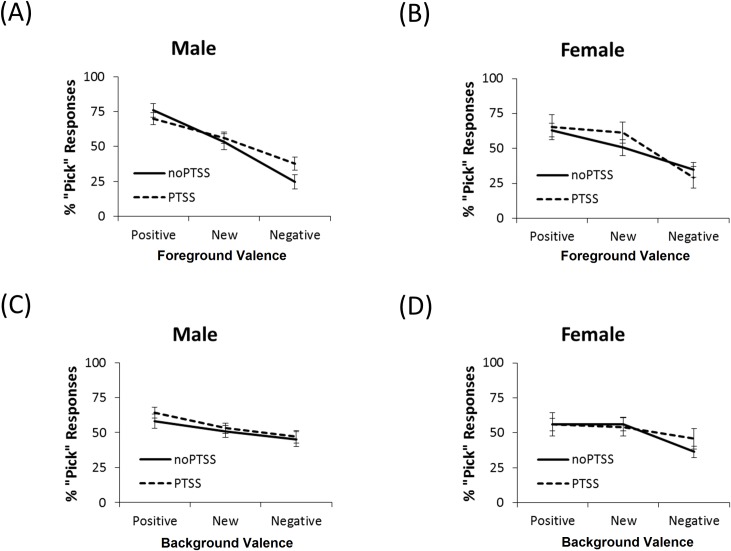
Percent “pick” responses as a function of gender, PTSS, foreground and background valence. (A) The noPTSS males show a decrease in “pick” responses from positive, to new, to negative foregrounds. PTSS males were similar, except that the difference between responses to positive and new foregrounds did not reach significance. (B) Both noPTSS and PTSS females picked positive and new foregrounds more often than negative foregrounds. (C) Further examination of background valence effects indicated that, among noPTSS males, there was no significant effect of background on responding, although in PTSS males, the difference between responding to positive and negative backgrounds was significant. This effect, however, was small. (D) The noPTSS females made significantly more “pick” responses with either the positive or new backgrounds compared to the negative background. In contrast, PTSS females showed no effect of background on responding.

Finally, there were three-way interactions between foreground valence, PTSS and gender *F*(2,232) = 4.48, *p* = .012, and background valence, PTSS and gender *F*(1.76,204.57) = 5.61, *p* = .004. To follow up on these interactions, we first considered responding to positive, negative, and new foregrounds, regardless of background. In noPTSS males (Fig 7A), responding to positive foregrounds was greater than to new foregrounds, and both were greater than negative foregrounds. The same pattern held for males with PTSS (all *t*>3.7, all *p*≤0.001, except PTSS males positive vs. new, which fell short of corrected significance, *t*(48) = 2.48, *p* = .017). However, in both noPTSS and PTSS females (Fig 7B), the pattern was to treat positive and new foregrounds as similar (both *t*<2, both *p*>.100) but respond significantly less to negative foregrounds (all *t*>3, *p* < .008, except new vs. negative in noPTSS females, *t*(31) = 2.29, *p* = .029).

Three independent-samples *t*-tests were also run to compare males and females at each foreground valence (ignoring PTSS). None of these comparisons approached significance (positive foreground, *t*(126) = 1.58, p = .116; new foreground, *t*(126) = 0.07, *p* = .946; negative foreground, *t*(126) = 0.01, *p* = .996). In addition, three ANOVAs were run to examine the interaction between gender and PTSS on responding to either positive, new or negative foregrounds. In all three cases, there were no significant main effects of gender or PTSS, and no significant interactions (all *p*>.100).

Next, we examined the interaction with background, regardless of foreground. Among noPTSS males, there were no significant effects of background (paired-samples *t*-tests, all t<2, all p>.050). With PTSS males, this changed so that there was significantly more responding in the positive than negative background (*t*(48) = 3.22, *p* = .002) and a trend for more responding in the positive than new background (*t*(48) = 2.57, *p* = .013), although as Fig 7C shows, the effect size was small. On the other hand, among noPTSS females (Fig 7D), there was significantly more responding in either the positive or new backgrounds than in the negative background (all t>3, all p≤.003). Responding in the positive and new backgrounds did not differ (t(31) = 0.07, p = .948). With PTSS, the pattern changed so that there were no effects of background (all *t*<1.5, all *p*>.100). Thus, while females in the noPTSS group appeared sensitive to the background, there was no evidence for this in PTSS females.

As above, three independent-samples *t*-tests comparing males and females at each background valence (positive, new or negative) were also performed. None of the comparisons were significant (all *p*>.100). Second, three ANOVAs examined the interaction between gender and PTSS on responding to either the positive, new or negative backgrounds. There were no significant main effects, and no significant interactions (all *p*>.100).

Finally, to check whether these group differences were driven by biases to make “pick” vs. “skip” responses, univariate ANOVA was conducted on overall percent “pick” responses across the entire testing phase. This analysis revealed no significant differences as a function of group, gender, or PTSS (all *p*>.200).

### Generalization task: Confidence ratings

**Fig 8:**
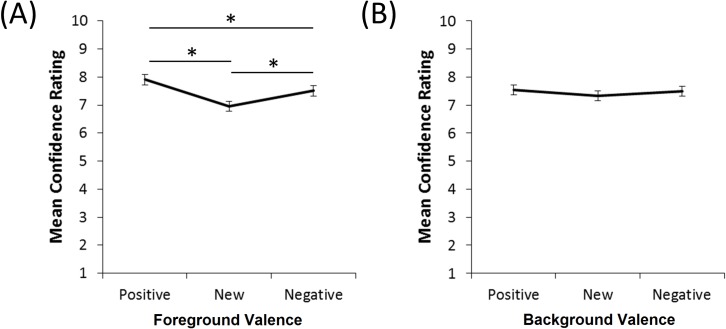
Confidence ratings. (A) Participants were significantly more confident in their responses to positive and negative objects than to new objects. (B) Confidence ratings did not differ as a function of contextual valence. Asterisks indicate significant within-subject differences.

Turning to confidence ratings, mixed ANOVA with within-subject factors of object valence (3 levels) and context valence (3 levels), and between-subject factors of gender, PTSS and group, revealed a small, but significant effect of object valence (*F*(1.78, 206.95) = 8.77, *p* < .001). Specifically, as shown in Fig 8, regardless of context, participants tended to be more confident in their responses to stimuli with positive objects than new objects (paired-samples *t*-test, *t*(127) = 6.56, *p* < .001) or negative objects (*t*(127) = 3.80, *p* < .001), and more confident in their responses to stimuli with negative objects than new objects (*t*(127) = 3.69, *p* < .001). There was also an interaction between object valence, group, and PTSS (*F*(3.57, 206.95) = 2.96, *p* = .025). There were no other effects or interactions (all *p*>.05). Post-hoc Bonferroni-corrected tests (alpha adjusted to .05/3 = .0167) to follow up on the significant interaction found a trend for reduced confidence ratings by the PTSS than noPTSS, but only for the combat-exposed veteran group. However, this fell short of corrected significance (p = .019).

Average confidence rating across all transfer trials was 7.5, SD 1.8. The average confidence rating did not differ as a function of group, gender, or PTSS status (univariate ANOVA, all *F*<2.5, all *p*>.100). However, average confidence ratings were significantly correlated with training phase performance, indexed by total percent correct responses over the 64 training trials (Pearson’s *r* = .228, *p* = .010).

## Discussion

This study compared a large sample of individuals including males and females, veterans and civilians, both with and without severe PTSD symptoms, on a task designed to assess generalization when positive, negative, or new foregrounds are presented in positive, negative, or new contexts. Overall, both foreground and background modulated responding, but this depended on PTSD symptom burden and gender. Below, these results are discussed further, and related to prior studies.

### Reward- and punishment-based learning

PTSS did not affect the training phase, consistent with a prior study by Levy-Gigi et al. [15], which also found no difference between individuals diagnosed with PTSD and healthy controls on a similar deterministic task. Thus, the emerging conclusion from these studies is that individuals with either PTSD or subclinical symptoms are not necessarily abnormal at either learning to obtain reward or to avoid punishment when cue-outcome mappings are deterministic and reinforcement can be accurately predicted. Instead, a previous study by Myers et al. [25] reported that individuals with more severe PTSD symptoms differ from healthy controls in how they process ambiguous feedback when cue-outcome mappings are probabilistic and expectancies can be violated. Taken together, these results may be important for understanding how particular symptoms emerge in PTSD, and in optimizing treatment.

In the current study, punishment was weighted more heavily than reward, which should have accelerated learning on punishment trials. Surprisingly, participants learned to “pick” faster on reward trials than they learned to “skip” on punishment trials. The early bias for “pick” responses (Fig 4) might reflect an intolerance of uncertainty, previously linked to PTSD symptoms [26], biasing participants away from “skip” responses. However, by the second training block, performance was similar on both types of trial, with an approximate balance between “pick” and “skip” responses. Therefore, the earlier tendency may have been because subjects were still exploring the stimulus-outcome contingencies, and “pick” responses provided informative feedback. Nonetheless, it remains unknown how the current results would have been affected if both reward and punishment were weighted similarly.

### Generalization

After learning to “pick” on trials with positively-valenced, and “skip” on trials with negatively-valenced configurations of foregrounds and backgrounds, participants were presented with various new combinations of cues and asked whether they would “pick” or “skip” each. As shown in Fig 5, the task was successful in eliciting responses modulated by both the reinforcement history of the foreground and of the background. In particular, stimuli containing positive foregrounds were picked more often than those containing negative foregrounds, regardless of background, while stimuli with foregrounds placed against positive backgrounds were picked more often than those containing negative backgrounds, regardless of the foreground. Stimuli containing novel elements tended to generate intermediate levels of “pick” responses. Importantly, all stimuli in this phase involved new configurations, even if both the foreground and background had been previously experienced during the training phase. Nevertheless, as shown in Fig 5A, subjects tended to “pick” stimuli where both cues were positive about 75% of the time, and to “skip” stimuli where both cues were negative about 75% of the time–approximately the same rate of responding as to the familiar configurations at the end of training block 4.

In contrast to what might be expected based on the prior Levy-Gigi et al. [14] study, the current study found no evidence that the PTSS group was less likely than the noPTSS group to “pick” new foregrounds when they were presented against a negative background. Thus, the current results suggest that it is not the novelty of the foreground, but rather differences in the processing of the background–particularly, in distinguishing new versus negative backgrounds–that drive group differences. Specifically, while all subjects tended to “pick” more when the background was positive than when it was negative, the noPTSS group was also more likely to “pick” new relative to negative backgrounds. In contrast, the PTSS group did not show this difference.

A possible interpretation of this finding is that the noPTSS group distinguished between novel and negative backgrounds whereas the PTSS group did not. This is consistent with an altered ability to process cue configurations to disambiguate the meaning of any given cue in PTSD. In particular, this may be the case when learning that cues experienced in an unfamiliar configuration do not have the same meaning as when they were previously experienced in the context of an aversive event (e.g. such as the trauma). In turn, this may lead to increased generalization and subsequent re-experiencing. Alternatively, this result may reflect a loss of contextual information, which would similarly lead to overgeneralization. However, it is important to note that in the current study, the background component of each stimulus varied from trial to trial, and may therefore not be treated as a context, which usually consists of cues that remain constant throughout learning. Instead, the background may have simply represented a less salient component of the stimulus. Consistent with this, the smaller effect size of background valence compared to foreground valence in Fig 5B and 5C, may reflect lower attention paid to the background component of stimuli, and thus reduced strength of learned associations involving that component.

The current study also found gender differences in how the background was processed. Specifically, Fig 7 suggests that females tend to treat positive and new backgrounds as similar to each other, but different from negative backgrounds, whereas males were more likely to treat new backgrounds as similar to negative backgrounds. There were also interactions between PTSD symptoms and gender, which appeared to reflect a reduction of background processing in females with PTSS compared to those in the noPTSS group. Given the fact that females are at greater for PTSD than males, and the potential importance of overgeneralization in PTSD, it is perhaps surprising that more attention has not been paid to gender differences in similar tasks, or more generally, in associative learning tasks. The current results suggest that increased generalization may be an important correlate of PTSD symptoms in females, but less so in males. This conclusion remains tentative, based only on a single experimental paradigm and reflected in a fairly small effect size. Still, the issue appears worthy of further investigation, since it might help elucidate not only the cognitive processing substrates driving gender differences in PTSD risk, but also potential pathways to remediate symptoms that might be differentially effective in females vs. males.

Lastly, participants were asked to provide confidence ratings, as well as behavioral responses, during the transfer phase. In general, participants appeared fairly confident of their responses. Unsurprisingly, confidence was greater for trials with a familiar (positive or negative) than with a novel object. There was no such effect of background on confidence, despite that the background did modulate responding, as evidenced by the behavioral results. This might suggest that, at a conscious or explicit level, participants believed that they were basing responses on foreground history, and that background may have influenced responding at a more implicit level. In any case, there was no significant effect of PTSS or gender on confidence ratings, indicating that groups were equivalently confident in the accuracy of their ability to predict outcomes to new cue configurations.

### PTSD symptoms in civilian and veteran samples

In the current sample, about 38% of veteran participants reported history of exposure to combat, and about 18% were female, which is similar to the distribution in prior studies drawing samples from the VA New Jersey Health Care System population [23, 25, 27]. As expected, few combat veterans were female.

Rates of PTSS assessed by PCL-C were nearly 60% in the combat veterans and over 75% in combat veterans. This rate is considerably higher than the 15–20% suggested by some prior epidemiological studies of PTSD in veterans [2, 3, 28]. The higher rate observed here is likely due to reliance on self-report to assess PTSD symptom burden, but may also reflect recruitment from a VA medical center where the number of individuals with significant health issues is higher than in the general population, in addition to self-selection bias based on interest to volunteer to participate in a research study related to PTSD. Those who meet symptom criteria as assessed by PCL do not necessarily meet full diagnostic criteria for PTSD, which (under the current *DSM-5* definition) includes additional considerations related to trauma exposure, duration of symptoms, and impact on daily life [29]. Even so, the high rate of symptoms in the current sample is generally consistent with the concept of widespread subclinical PTSD among veterans, and emphasizes the need to consider the negative impact subclinical symptoms can have on daily life and health outcomes. Additionally, although combat exposure is considered a risk for PTSD [28, 30, 31], the high rate of PTSS among non-combat veterans in the current sample highlights the fact that other military-related events, such as deployment and wartime experiences, as well as non-military traumas, are also important in assessing PTSD risk among veterans. The current study found little evidence of differences between veterans as a function of combat exposure, in either rates of PTSS or in learning and generalization.

The current civilian sample also had a high rate of participants meeting PTSS criteria, amounting to over 20% of the group. While this rate was significantly lower than in the veteran groups, it is higher than the expected lifetime prevalence of PTSD for the general US population, estimated at about 7% [1]. The higher rates observed here could again reflect self-selection bias among volunteers, but also that civilian participants (like our veteran samples) were largely African American and were recruited from an urban environment. Prior studies have suggested PTSD rates of 20–34% among African Americans or participants recruited from urban settings [32–34], although only a fraction of these individuals may seek or receive treatment. These rates are consistent with those observed in the current civilian sample.

Although prior studies have often documented higher rates of PTSD and higher PCL scores in females [1], no gender differences in PCL scores or PTSS rates were observed in our study. This may simply be due to the high inclusion of PTSS individuals in all three groups. Nevertheless, the lack of a gender effect in PTSS in the current study made it possible to examine effects of gender and PTSS separately in the analysis of behavioral results.

### Limitations and open questions

The current study had several limitations. First, although the study was designed to examine possible main effects of PTSS and gender, both variables were imbalanced in the civilian vs. veteran groups, with relatively few combat-exposed female veterans, and more PTSS cases among veterans than civilians. These patterns were not unexpected but do limit the ability to draw strong conclusions about interactions involving group, which may be accounted for by differences in PTSD symptom severity rather than group membership. Nevertheless, there was little evidence for qualitative differences in behavior in veterans vs. civilians, or between combat and non-combat veterans. A potentially important factor, which was not considered in the current study, is time since last combat experience. Combat veterans who served in Vietnam were overrepresented in the current sample and it remains unknown how this may have influenced the results.

The study was also designed to group participants based on self-assessed severity of PTSD symptoms, and these individuals may differ in important ways from patients who have undergone clinical assessment to determine diagnostic status. While PCL is used widely in clinical settings to aid diagnosis and track symptoms, and validation studies have repeatedly shown that the PCL has high internal validity and correlates highly with clinician-assessed PTSD [35–38]. The noPTSS group included individuals who reported at least some PTSD symptoms, who may also differ in important ways from healthy controls with no PTSD symptoms. Accordingly, future studies could examine this task in patients diagnosed with PTSD and in controls with no PTSD symptoms. Presumably, this could also boost effect size by increasing heterogeneity between groups. Additionally, the version of the PCL used here (PCL-C) was originally designed to include questions corresponding to *DSM-IV* symptom criteria [20], and these criteria have changed somewhat with the shift to the current edition–the *DSM-5* [29].

It is also worth noting that relatively few participants reached optimal performance during the training phase, and that group performance (as illustrated in Fig 3A) only approached about 75% by the end of training, compared with a theoretical maximum of 100%. This poor performance is surprising given that there were only four stimuli and that outcomes were deterministic, rather than probabilistic, as in a prior version of this task [25]. Although data from a number of participants were dropped due to apparent non-compliance, the remainder appeared motivated to complete the task, and as shown in Fig 3C and 3D, by the end of training, most were performing above chance on both reward-and punishment-based trials. Even so, not all participants were equally accurate on both trial types. This individual variability in learning was observed previously [25], and appears to reflect individual differences in whether participants interpret the no-feedback outcome as signaling reward (successful avoidance of punishment), which facilitates learning on punishment trials but potentially impairs learning on reward trials, or as signaling punishment (missed opportunity for reward), which facilitates learning on reward trials but potentially impairs learning on punishment trials. Still, it is possible that results would be different if more subjects had perfectly mastered the initial training. This remains an open question for future research. Another limitation of the current study is that IQ was not directly measured, which could be an important source of variation in learning performance. On the other hand, level of education did not differ between groups in the current sample, and previous studies found no differences in estimated IQ between participants with and without PTSD [14, 15].

Lastly, a key question for understanding learning and generalization in PTSD is whether differences in generalization exist prior to trauma exposure and increase the risk to develop PTSD, or are symptoms that emerge only after the fact. This question can only be addressed by longitudinal studies. If altered generalization exists prior to trauma then this could be potentially important in screening for PTSD risk in trauma-exposed individuals. On the other hand, if it is a symptom, then tasks such as the current paradigm which assess generalization could be useful tools to track symptom development and, hopefully, response to therapy.

## Supporting information

S1 File Supplemental materials. Values of the means and standard errors used to construct data figures.
(XLSX)
